# EVIDENCE-BASED REVIEWS & RECOMMENDATIONS FROM THE BRAZILIAN SOCIETY OF SHOULDER AND ELBOW SURGERY: HYALURONIC ACID INJECTION VERSUS PHYSIOTHERAPY FOR ROTATOR CUFF TENDINOPATHY IN ADULTS

**DOI:** 10.1590/1413-785220263401e308948

**Published:** 2026-08-31

**Authors:** Marcos Rassi Fernandes, Leonardo Zanesco, Joel Murachovsky, Guilherme Grisi Mouraria, Paulo Henrique Schmidt Lara, Paulo Santoro Belangero, João Felipe de Medeiros, Eduardo Angeli Malavolta

**Affiliations:** 1 Universidade Federal de Goias, Faculdade de Medicina, Goiania, GO, Brazil.; 2 Universidade de Sao Paulo, Faculdade de Medicina, Hospital das Clinicas HCFMUSP, Sao Paulo, SP, Brazil.; 3 Faculdade de Medicina do ABC, Santo Andre, SP, Brazil.; 4 Universidade Estadual de Campinas, UNICAMP, Campinas, SP, Brazil.; 5 Escola Paulista de Medicina, UNIFESP, Sao Paulo, SP, Brazil.; 6 Universidade Federal do Rio Grande do Norte, Natal, RN, Brazil.

**Keywords:** Rotator Cuff Tendinopathy, Injections, Intra-articular, Physiotherapy, Shoulder Pain, Randomized Controlled Trials, Systematic Review, Tendinopatia do Manguito Rotador, Injeções Intra-articulares, Fisioterapia, Dor no Ombro, Ensaios Clínicos Randomizados, Revisão Sistemática

## Abstract

**Introduction:**

Rotator cuff tendinopathy is a common cause of shoulder pain and disability, and the optimal nonoperative strategy remains debated. Physiotherapy is the mainstay of initial management, whereas hyaluronic acid injection has been proposed as an alternative or adjunct; their comparative effectiveness is uncertain.

**Methods:**

An evidence-based systematic review was conducted in PubMed, Embase, Cochrane Library, and the Regional Portal of the Virtual Health Library (BVS) up to February 2026. Randomized controlled trials comparing hyaluronic acid injection with physiotherapy in adults with rotator cuff tendinopathy were included. Outcomes were pain, function, range of motion, and quality of life. Certainty of evidence was assessed with the GRADE approach.

**Results:**

Two randomized controlled trials (130 participants) met the eligibility criteria. Hyaluronic acid injection was associated with greater short-term improvement in pain, range of motion, and quality of life than physiotherapy, particularly within the first three months. Functional outcomes were inconsistent: one trial reported comparable improvement between groups, whereas the other favored hyaluronic acid. Certainty of evidence was very low because of small sample sizes, risk of bias, and imprecision.

**Conclusion:**

Very low-quality evidence suggests that hyaluronic acid injection may provide superior short-term benefits over physiotherapy for rotator cuff tendinopathy, mainly up to three months. The available evidence remains limited and at high risk of bias; further high-quality studies are required. ***Level of evidence II; Systematic review.***

## RESUMO

**Introdução:**

A tendinopatia do manguito rotador é causa frequente de dor e incapacidade do ombro, e o tratamento não operatório ideal permanece controverso. A fisioterapia é o pilar do manejo inicial, enquanto a infiltração com ácido hialurônico tem sido proposta como alternativa ou adjuvante; sua efetividade comparativa é incerta.

**Métodos:**

Foi realizada revisão sistemática baseada em evidências nas bases PubMed, Embase, Cochrane Library e Portal Regional da Biblioteca Virtual em Saúde (BVS) até fevereiro de 2026. Foram incluídos ensaios clínicos randomizados que compararam a infiltração com ácido hialurônico e a fisioterapia em adultos com tendinopatia do manguito rotador. Os desfechos avaliados foram dor, função, amplitude de movimento e qualidade de vida. A certeza da evidência foi avaliada por meio da abordagem GRADE.

**Resultados:**

Dois ensaios clínicos randomizados (130 participantes) preencheram os critérios de elegibilidade. A infiltração com ácido hialurônico associou-se a maior melhora de curto prazo da dor, da amplitude de movimento e da qualidade de vida em comparação com a fisioterapia, especialmente até três meses. Os desfechos funcionais foram inconsistentes: um ensaio relatou melhora semelhante entre os grupos, enquanto o outro favoreceu o ácido hialurônico. A certeza da evidência foi muito baixa em razão do reduzido tamanho amostral, do risco de viés e da imprecisão.

**Conclusão:**

Evidência de muito baixa qualidade sugere que a infiltração com ácido hialurônico pode oferecer benefícios superiores no curto prazo em relação à fisioterapia para a tendinopatia do manguito rotador, sobretudo até três meses. A evidência disponível é limitada e de alto risco de viés, sendo necessários estudos de melhor qualidade. ***Nível de evidência II; Revisão sistemática.***

## INTRODUCTION

Rotator cuff tendinopathy is a broad term describing degenerative or inflammatory changes of the rotator cuff tendons that do not necessarily involve a full-thickness tear. It may present with degeneration of the tendon fibers, collagen disorganization, tendon thickening or edema, and inflammation.^1,2^

Management of rotator cuff tendinopathy depends on the intensity of symptoms, the thickness of the tendon lesion, and the patient's age and functional demand. Physiotherapy is the most widely used treatment for patients with tendinopathy or partial-thickness rotator cuff tears involving less than 50% of the tendon thickness.^3,4^

Subacromial, peritendinous, or intratendinous injection of hyaluronic acid (HA) may be used in the management of rotator cuff tendinopathy. The proposed effects of this agent include improvement of the viscoelasticity of the synovial fluid, reduction of friction between the tendon and adjacent structures, decreased inflammatory mediators, inhibition of metalloproteinases, possible stimulation of tenocyte proliferation, and reduction of local sensitivity and pain.^5,6^

Some adverse effects of HA injection have been reported, including transient local pain, foreign-body reaction, pyarthritis, cellulitis, mild inflammatory reaction, and infection.^5,7^ Given these considerations, a direct comparison of functional and pain outcomes between HA injection and conventional physiotherapy is warranted.

This evidence-based review was developed within a collaborative initiative of the Brazilian Society of Shoulder and Elbow Surgery (SBCOC) Consensus Project, which aims to synthesize the best available evidence using rigorous, transparent methods and to translate it into clear, practical recommendations for clinical practice.

This study was conducted at the initiative of the Brazilian Society of Shoulder and Elbow Surgery with the participation of Cochrane Brazil.

### Research Question

Is hyaluronic acid injection more effective than physiotherapy for the treatment of rotator cuff tendinopathy in adults?

## METHODS

- Databases searched: PubMed, Embase, Cochrane Library, and BVS.
- Descriptors: As described in Appendix 1.
- Search period: Until February 2026.
- Study selection: Updated systematic reviews (SRs) with adequate methodological quality were prioritized. In the absence of SRs, randomized controlled trials (RCTs) and cohort studies were considered.

## RESULTS

**Figure 1:**
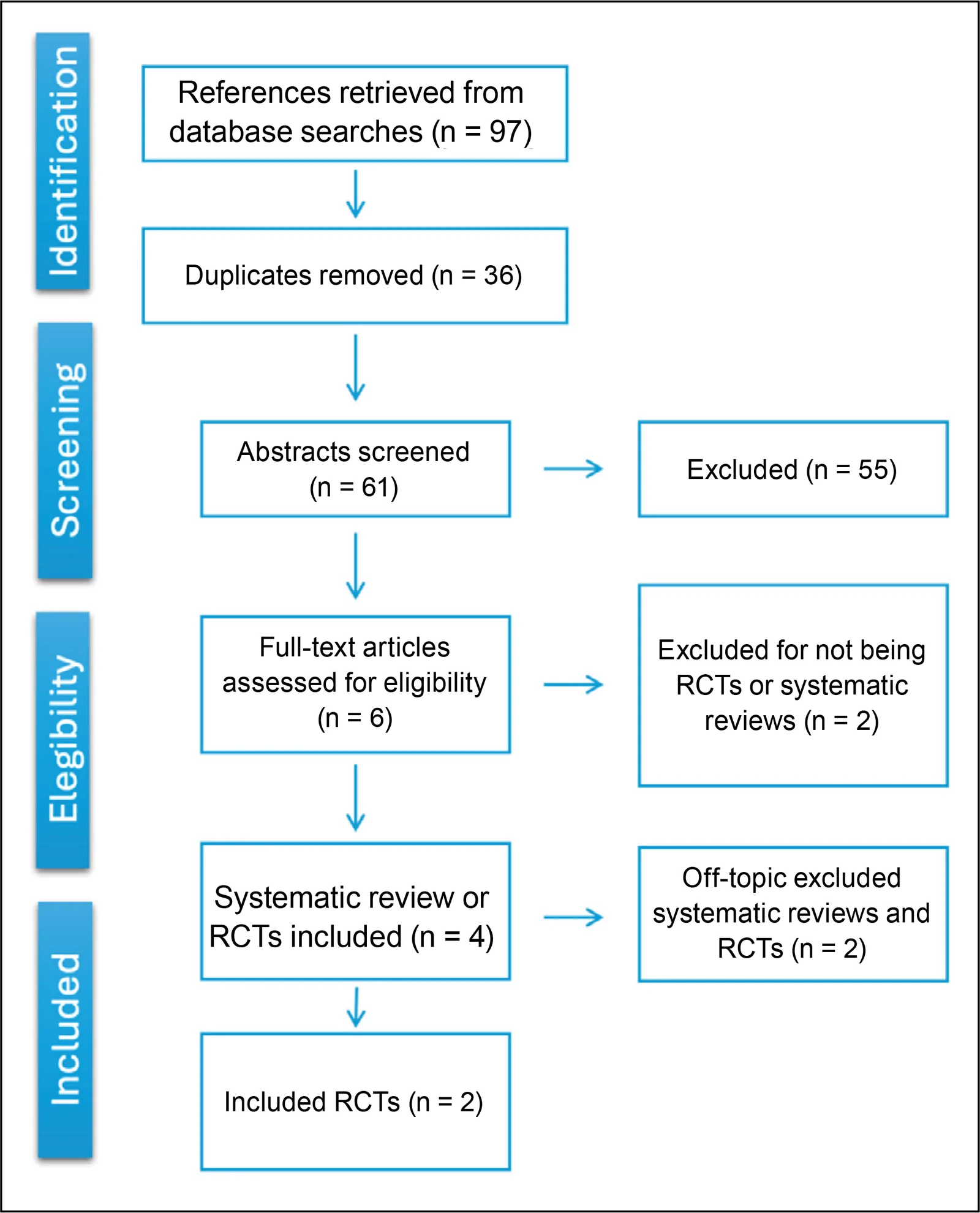
Flow diagram of the literature search.

The search retrieved 97 references (22 in PubMed, 7 in Embase, 53 in the Cochrane Library, and 15 in the Regional Portal BVS). Thirty-six records were removed as duplicates. After screening the abstracts of the remaining 61 studies, 55 were excluded. Of the six retained for full assessment, two were discarded for not being SRs or RCTs. Two systematic reviews were subsequently excluded: the study by Goulart et al.^8^, which evaluated only comparisons between injection types or placebo, and that by Giovannetti de Sanctis et al.^9^, in which the injected agent was a corticosteroid and no comparison with physiotherapy was included. Two RCTs therefore remained (Figure 1).

### Included studies

Few studies in the literature directly compared the effectiveness of HA injection with physiotherapy. No systematic review performed this direct comparison; only two RCTs were identified.

Rezasoltani et al.^10^ evaluated 51 participants aged 20 to 55 years with supraspinatus tendinopathy, randomized to subacromial HA injection (n = 28) or physiotherapy (n = 23). The HA used had a molecular weight of 500–700 kDa, and the rehabilitation protocol comprised three physiotherapy sessions per week for three months—a total of 36 sessions of rotator cuff strengthening exercises. The outcomes assessed were pain (Visual Analogue Scale, VAS), range of motion, shoulder functional disability, and quality of life (World Health Organization questionnaire), evaluated at one, four, and twelve weeks. The authors concluded that HA was more effective than physiotherapy for pain control and for improvement of range of motion and quality of life up to three months of follow-up, whereas both approaches reduced functional disability to a similar degree.

Esmaily et al.^11^, in an RCT including 79 participants with rotator cuff tendinopathy, compared low- and high-molecular-weight HA with physiotherapy. Participants were allocated to three groups: low-molecular-weight HA (n = 28), high-molecular-weight HA (n = 27), and physiotherapy (n = 24). The injections were administered into the subacromial space at a dose of 20 mg, and the physiotherapy group performed 10 exercise-based sessions. The primary outcome was shoulder pain; secondary outcomes were the DASH score, range of motion, and quality of life, assessed at baseline and after one and three months, with pain additionally evaluated at six months. The authors concluded that HA injections were more effective in controlling pain, reducing disability, increasing range of motion, and improving quality of life. No difference in effectiveness was found between the two molecular weights; however, the low-molecular-weight group reported less intense local pain than the high-molecular-weight group. The clinical benefits of HA persisted for at least three months, and pain relief was partially maintained up to six months.

The general characteristics of the included studies and the certainty of evidence according to the Grading of Recommendations Assessment, Development and Evaluation (GRADE) approach are presented in Tables 1 and 2, respectively.^12,13^

**Table 1:** Characteristics of the included studies.

Included studies	Study design	Sample size	Previously published protocol
Rezasoltani Z. et al.^10^	RCT	51	Not published
Esmaily H. et al.^11^	RCT	79	Not published

RCT: randomized controlled trial.

**Table 2:** Quality of evidence (GRADE): hyaluronic acid injection versus physiotherapy.

Outcome	Studies (N)	Certainty of evidence
Pain	2 studies (130)	Very low^1^,^2^,^3^
Shoulder function (various scores)	2 studies (130)	Very low^1^,^2^,^3^

1 Limited sample size;

2 Imprecision (wide confidence interval);

3 Risk of bias of the included studies;

4 Lack of a prior protocol.

Note: GRADE (Grading of Recommendations Assessment, Development and Evaluation) is a tool used to assess and grade the certainty of evidence. It is based on five domains: risk of bias, imprecision, inconsistency, indirectness, and publication bias. The certainty of evidence is classified as very low, low, moderate, or high.

### Study Limitations

Further RCTs of higher methodological quality are needed to compare HA injection with physiotherapy for the treatment of rotator cuff tendinopathy. Such studies should be multicenter, include a larger number of physiotherapy sessions, enroll larger samples, and provide a minimum follow-up of two years.

### Recommendation

There is evidence, although of very low quality, that hyaluronic acid injection in the shoulder is more effective than physiotherapy in the short term for the treatment of rotator cuff tendinopathy in adults, with respect to pain and function. This benefit is most evident within the first three months of treatment.

The SBCOC Consensus Project recommends that the surgeon individually assess the indication for hyaluronic acid treatment in patients with rotator cuff tendinopathy. Physiotherapy remains the initial treatment of choice. Factors such as functional demand, rehabilitation time, and the expected time to return to work and/or sport should be considered when indicating hyaluronic acid treatment and its combination with physiotherapy.

## CONCLUSION

There is very low-quality evidence demonstrating that hyaluronic acid injection is more effective than physiotherapy for the treatment of rotator cuff tendinopathy in adults, particularly for short-term pain and function up to three months.

## Data Availability

The contents underlying the research are available in the manuscript.

## Appendix 1 Database search strategies

**Table:**

**PUBMED**
#1 "Rotator Cuff Injuries"[Mesh] OR (Cuff Injury, Rotator) OR (Injury, Rotator Cuff) OR (Rotator Cuff Injury) OR (Rotator Cuff Tears) OR (Rotator Cuff Tear) OR (Tear, Rotator Cuff) OR (Tears, Rotator Cuff) OR (Rotator Cuff Tendinitis) OR (Rotator Cuff Tendinitides) OR (Tendinitis, Rotator Cuff) OR (Rotator Cuff Tendinosis) OR (Rotator Cuff Tendinoses) OR (Tendinoses, Rotator Cuff) OR (Tendinosis, Rotator Cuff) OR (Glenoid Labral Tears) OR (Glenoid Labral Tear) OR (Labral Tear, Glenoid) OR (Labral Tears, Glenoid) OR (Tear, Glenoid Labral).
#2 "Hyaluronic Acid"[Mesh] OR (Acid, Hyaluronic) OR (Amvisc) OR (Luronit) OR (Hyvisc) OR (Hyaluronan) OR (Sodium Hyaluronate) OR (Hyaluronate, Sodium) OR (Hyaluronate Sodium) OR (Healon) OR (Etamucine) OR (Amo Vitrax) OR (Vitrax, Amo) OR (Biolon).
#3 "Injections, Intra-Articular"[Mesh] OR (Injection, Intra-Articular) OR (Intra-Articular Injection) OR (Intra Articular Injection) OR (Articular Injection, Intra) OR (Articular Injections, Intra) OR (Injection, Intra Articular) OR (Injections, Intra Articular) OR (Intra Articular Injections) OR (Intraarticular Injection) OR (Injection, Intraarticular) OR (Intra-Articular Injections) OR (Injections, Intraarticular) OR (Intraarticular Injections).
#4 "Physical Therapy Modalities"[Mesh] OR (Modalities, Physical Therapy) OR (Modality, Physical Therapy) OR (Physical Therapy Modality) OR (Physical Therapy Techniques) OR (Physical Therapy Technique) OR (Techniques, Physical Therapy) OR (Physiotherapy (Techniques)) OR (Physiotherapies (Techniques)) OR (Neurological Physiotherapy) OR (Physiotherapy, Neurological) OR (Neurophysiotherapy) OR (Group Physiotherapy) OR (Group Physiotherapies) OR (Physiotherapies, Group) OR (Physiotherapy, Group) OR (Physical Therapy) OR (Physical Therapies) OR (Therapy, Physical).
#5 #2 AND #3.
#6 #1 AND #5 AND #4 — 22 records.

**Table:**

**COCHRANE LIBRARY**
#1 (Rotator Cuff Injuries) OR (Cuff Injury, Rotator) OR (Injury, Rotator Cuff) OR (Rotator Cuff Injury) OR (Rotator Cuff Tears) OR (Rotator Cuff Tear) OR (Tear, Rotator Cuff) OR (Tears, Rotator Cuff) OR (Rotator Cuff Tendinitis) OR (Rotator Cuff Tendinitides) OR (Tendinitis, Rotator Cuff) OR (Rotator Cuff Tendinosis) OR (Rotator Cuff Tendinoses) OR (Tendinoses, Rotator Cuff) OR (Tendinosis, Rotator Cuff) OR (Glenoid Labral Tears) OR (Glenoid Labral Tear) OR (Labral Tear, Glenoid) OR (Labral Tears, Glenoid) OR (Tear, Glenoid Labral).
#2 (Hyaluronic Acid) OR (Acid, Hyaluronic) OR (Amvisc) OR (Luronit) OR (Hyvisc) OR (Hyaluronan) OR (Sodium Hyaluronate) OR (Hyaluronate, Sodium) OR (Hyaluronate Sodium) OR (Healon) OR (Etamucine) OR (Amo Vitrax) OR (Vitrax, Amo) OR (Biolon).
#3 (Injections, Intra-Articular) OR (Injection, Intra-Articular) OR (Intra-Articular Injection) OR (Intra Articular Injection) OR (Articular Injection, Intra) OR (Articular Injections, Intra) OR (Injection, Intra Articular) OR (Injections, Intra Articular) OR (Intra Articular Injections) OR (Intraarticular Injection) OR (Injection, Intraarticular) OR (Intra-Articular Injections) OR (Injections, Intraarticular) OR (Intraarticular Injections).
#4 (Physical Therapy Modalities) OR (Modalities, Physical Therapy) OR (Modality, Physical Therapy) OR (Physical Therapy Modality) OR (Physical Therapy Techniques) OR (Physical Therapy Technique) OR (Techniques, Physical Therapy) OR (Physiotherapy (Techniques)) OR (Physiotherapies (Techniques)) OR (Neurological Physiotherapy) OR (Physiotherapy, Neurological) OR (Neurophysiotherapy) OR (Group Physiotherapy) OR (Group Physiotherapies) OR (Physiotherapies, Group) OR (Physiotherapy, Group) OR (Physical Therapy) OR (Physical Therapies) OR (Therapy, Physical).
#5 #2 AND #3.
#6 #1 AND #5 AND #4 — 53 records.

**Table:**

**EMBASE**
#1 ‘rotator cuff injury’/exp OR ‘cuff injury’ OR ‘cuff lesion’ OR ‘rotator cuff disease’ OR ‘rotator cuff disorder’ OR ‘rotator cuff disorders’ OR ‘rotator cuff injuries’ OR ‘rotator cuff injury’ OR ‘rotator cuff lesion’ OR ‘rotator cuff lesions’ OR ‘rotator cuff pathology’ OR ‘rotator cuff tendinopathies’ OR ‘rotator cuff tendinopathy’ OR ‘shoulder rotator cuff degeneration’ OR ‘shoulder rotator cuff lesion’.
#2 ‘intraarticular drug administration’/exp OR ‘drug administration, intraarticular’ OR ‘injection, intraarticular’ OR ‘injections, intra-articular’ OR ‘intra-articular administration’ OR ‘intra-articular delivery’ OR ‘intra-articular drug administration’ OR ‘intra-articular drug delivery’ OR ‘intra-articular infusion’ OR ‘intra-articular injection’ OR ‘intra-articular injections’ OR ‘intra-articular medication’ OR ‘intra-articular treatment’ OR ‘intraarticular administration’ OR ‘intraarticular delivery’ OR ‘intraarticular drug administration’ OR ‘intraarticular infusion’ OR ‘intraarticular injection’ OR ‘intraarticular medication’ OR ‘intraarticular treatment’ OR ‘intracoxal drug administration’ OR ‘joint infusion’ OR ‘joint injection’ OR ‘treatment, intraarticular’.
#3 ‘hyaluronic acid’/exp OR ‘adant’ OR ‘amo vitrax’ OR ‘amvisc’ OR ‘artz’ OR ‘biolon’ OR ‘durolane’ OR ‘healon’ OR ‘hyalgan’ OR ‘hyaluronan’ OR ‘hyaluronate’ OR ‘hyaluronate sodium’ OR ‘hyaluronic acid’ OR ‘hylan g-f 20’ OR ‘monovisc’ OR ‘orthovisc’ OR ‘ostenil’ OR ‘sodium hyaluronate’ OR ‘supartz’ OR ‘suplasyn’ OR ‘synvisc’ (and related brand-name terms).
#4 ‘physiotherapy’/exp OR ‘physical therapy’ OR ‘physical therapy service’ OR ‘physical therapy techniques’ OR ‘physical treatment’ OR ‘physiotherapy’ OR ‘therapy, physical’.
#5 #2 AND #3.
#6 #1 AND #5 AND #4 — 27 records.
#7 #6 AND [embase]/lim NOT ([embase]/lim AND [medline]/lim) — 7 records.

**Table:**

**REGIONAL PORTAL (BVS)**
#1 MH:"Lesões do Manguito Rotador" OR (Rotator Cuff Injuries) OR (Roturas do Manguito Rotador) OR (Ruptura do Manguito Rotador) OR (Tendinite do Manguito Rotador) OR (Tendinose do Manguito Rotador) OR MH:C26.761.340$ OR MH:C26.803.063$ OR MH:C26.874.400$.
#2 MH:"Ácido Hialurônico" OR (Hyaluronic Acid) OR (Hialuronan) OR MH:D09.698.373.475$.
#3 MH:"Injeções Intra-Articulares" OR (Injections, Intra-Articular) OR (Injeção Intra-Articular) OR MH:E02.319.267.530.380$.
#4 MH:"Modalidades de Fisioterapia" OR (Physical Therapy Modalities) OR (Fisioterapia) OR (Técnicas de Fisioterapia) OR MH:E02.779$ OR MH:E02.831.535$.
#5 #1 AND #2 AND #3 AND #4 — 15 records.
